# Who are the best passing players in professional soccer? A machine learning approach for classifying passes with different levels of difficulty and discriminating the best passing players

**DOI:** 10.1371/journal.pone.0304139

**Published:** 2024-05-30

**Authors:** Murilo Merlin, Allan Pinto, Felipe Arruda Moura, Ricardo da Silva Torres, Sergio Augusto Cunha

**Affiliations:** 1 School of Physical Education, University of Campinas, Campinas, Brazil; 2 Faculty of São Vicente, São Vicente, Brazil; 3 Institute of Computing, University of Campinas, Campinas, Brazil; 4 Laboratory of Applied Biomechanics, State University of Londrina, Londrina, Brazil; 5 Faculty of Information Technology and Electrical Engineering, Department of ICT and Natural Sciences, NTNU–Norwegian University of Science and Technology, Ålesund, Norway; Universiti Malaysia Terengganu, MALAYSIA

## Abstract

The present study aimed to assess the use of technical-tactical variables and machine learning (ML) classifiers in the automatic classification of the passing difficulty (DP) level in soccer matches and to illustrate the use of the model with the best performance to distinguish the best passing players. We compared eight ML classifiers according to their accuracy performance in classifying passing events using 35 technical-tactical variables based on spatiotemporal data. The Support Vector Machine (SVM) algorithm achieved a balanced accuracy of 0.70 ± 0.04%, considering a multi-class classification. Next, we illustrate the use of the best-performing classifier in the assessment of players. In our study, 2,522 pass actions were classified by the SVM algorithm as low (53.9%), medium (23.6%), and high difficulty passes (22.5%). Furthermore, we used successful rates in low-DP, medium-DP, and high-DP as inputs for principal component analysis (PCA). The first principal component (PC1) showed a higher correlation with high-DP (0.80), followed by medium-DP (0.73), and low-DP accuracy (0.24). The PC1 scores were used to rank the best passing players. This information can be a very rich performance indication by ranking the best passing players and teams and can be applied in offensive sequences analysis and talent identification.

## Introduction

Analyzing soccer matches allows knowledge discovery that favors the planning and direction of training procedures to improve individual and collective performance. Positional and time data of players have provided a more contextual analysis of the match. In addition, the increase of sports-related data available in terms of volume, velocity, and variety of data—the big data characterization [1]–has required joint efforts from different areas, such as sports scientists and data scientists [2,3]. As a consequence, the application of machine learning (ML) and data mining (DM) techniques has increased considerably, with important contributions to performance analysis [4,5], injury prevention [6], strategy analysis [7], talent identification [2], and performance rating [8].

The pass is one of the most investigated technical elements of a match, which is considered a key performance indicator in soccer analysis [9,10]. Historically, the analysis of technical demand in soccer matches, especially the passing, has focused on inferences using frequency, density, order, and accuracy of actions [11–18].

More recent studies have used machine learning techniques for pass analysis. Examples include the prediction of passes based on concepts, such as risk and advantage of the passes [19]; estimation of time to intercept the ball from a pass [20]; assessment of the quality of passes [21]; and evaluation of passing effectiveness and involvement of players in creating scoring chances [22].

From our perspective, the pass is the basis of the soccer game. Soccer matches have become more complex, and faster, and players frequently need to work on reduced space to maintain ball possession [23]. The pass is the most used action by the player in ball possession, representing 69% of the ball actions [22]. On average, a typical match comprises 500 passes per team [24], i.e., a pass is performed every 10 seconds of a match. For each passing event, there is a different context, with different levels of difficulty, influenced by technical and tactical factors, based on the strategy of both teams.

Despite its relevance, current procedures for determining the pass performance of players or teams are limited. In practice, *high* performance is attributed to the player who achieves around 100% success rate when performing passes. But how challenging were the executed passes? One can argue that players who perform passes with a lower degree of difficulty are more likely to achieve better performance, and consequently to be considered the best passing players. The purpose of this study is precisely to contribute to address this limitation. We claim that, in order to have a fair indicator to analyze the passing performance, two challenges need to be faced. First, we need effective approaches for determining the level of difficulty of passes. Second, such approaches could be used to define performance indicators that consider not only the passing success rate but also the degree of difficulty of each executed pass.

In order to address the first challenge, we need an unambiguous definition of pass difficulty and to define which variables are relevant to characterize such difficulty. We consider the pass as a technical-tactical action that occurs at time and space, in which the difficulty of the action depends on the interaction of several technical characteristics (e.g., body position and orientation, ball contact, movement speed, and pass distance) and tactical (e.g., team interaction and space occupation by individual players, group, or by the team), to the ball reaches its destination. Therefore, the pass difficulty refers to the degree of technical and tactical demands that the passing player must complete the action successfully. Accurate positional data over time of each player, of both teams, allows the representation of these characteristics in a two-dimensional perspective. These characteristics (or variables) may serve as the basis for a classification system able to classify passes according to different levels of difficulty.

Regarding the second challenge, after classifying the passes according to the degree of difficulty, we need to determine the players’ performance considering the success rate in passes with different degrees of difficulty. We believe that a higher success rate in passes with a higher degree of difficulty should impact the most the passing performance indicator. This new procedure would, therefore, enable to distinguish and rank the best passing players and lead to relevant individual and collective performance indicators.

Therefore, the present study aimed: (i) to assess the use of technical-tactical variables and machine learning classifiers in the automatic classification of the passing difficulty level in soccer matches; (ii) to illustrate the use of the model with the best performance to distinguish the best passing players. Our hypothesis is that machine learning classifiers are effective in classifying the level of passing difficulty based on technical and tactical variable combination and that by classifying passes with different levels of difficulty we would be able to distinguish players and positions.

## Methods

**Fig 1:**
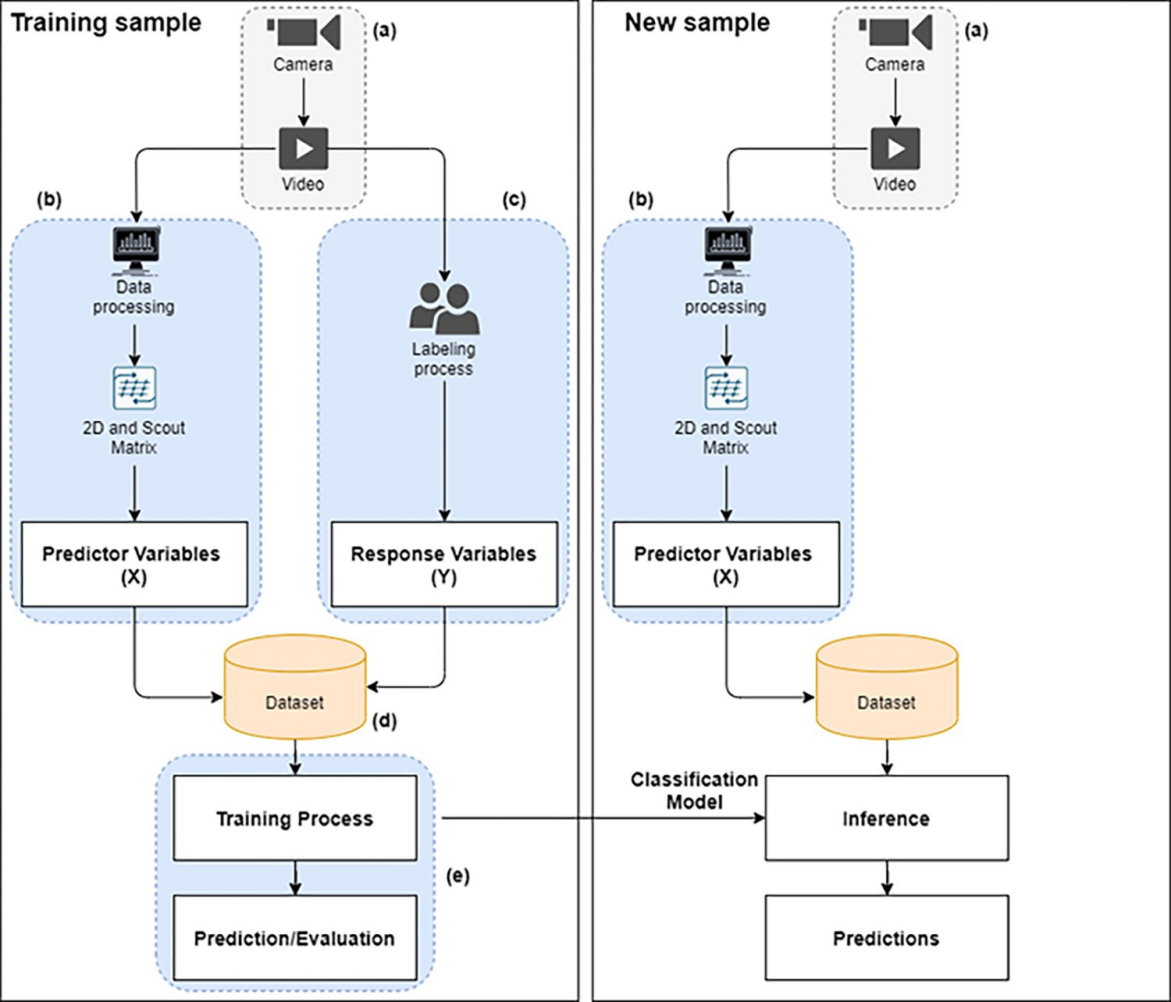
Study design started from data collection (a), going through the processing to obtain the 2D and scout matrix, and consequently obtaining the predictor variables (b). Then, the labeling process was carried out to obtain the variables’ responses (c) until the composition of the dataset (d). From the dataset, the training process and evaluation of the algorithms were performed to obtain the best classification model (e). This process was performed with part of the sample (training sample). Finally, the model was applied to classify automatically passes in unseen samples.

The present study first considered the five typical phases to build a classification model for automatically classifying pass actions according to their level of difficulty. Once generated, this model will be used to classify a new sample. The phases are organized as follows (Fig 1): a) Data collection and sample; b) Predictor variables; c) Response variables (Labeling process); d) Dataset; e) Supervised learning algorithms. The model with the best performance was applied to distinguish the best players and positions.

### Data collection and sample

We analyzed four official matches of the Brazilian Soccer Championship during the 2016 season. The four matches contained 2,522 passes in total. In our study, one sample refers to a pass. The matches were recorded by two digital cameras Sony CX405 Handycam® with Exmor R™ CMOS sensor, with an acquisition frequency of 30 Hz. In order to reduce the amount of data to be processed, the videos were reduced to 15 Hz by Virtual Dub software.

Subsequently, a semiautomatic tracking system was used to obtain the players’ 2D positional data using the software DVideo [25,26]. The players of each team were labeled as p = 1, 2,…, 14, including starting players and substitutes. Therefore, the 2D coordinates of each player (2D matrix) were defined as Xp(t) and Yp(t), where t represents each instant of time, and the X and Y axes represent the length and width of the pitch respectively. A Butterworth third-order low-pass digital filter with a cut-off frequency of 0.4 Hz was used according to previous study recommendations [27]. DVideo software has an automatic tracking rate of 94% of the processed frames, an average error of 0.3 m for the determination of player position, and an average error of 1.4% for the distance covered [28]. After smoothing, notational analysis was performed by an experienced operator to register the technical actions, synchronized with the positioning data [26].

### Predictor variables

Thirty-six predictor variables (*x*_1_,*x*_2_,…*x*_*n*_) were proposed for this study (Table 1). The variables were originally proposed, or based on similar previous studies [19,21,28,29] to build a multi-class classification model. All variables were obtained using the Matlab^®^2017 *software*.

**Table 1:** Tactical variables used and abbreviations, separated by groups.

Groups	Abbreviation	Variables (description)
**Passing player variables**	Nearest opp. PP_t0_	Distance between passing player and his nearest opponent at passing moment (t0).
	Density PP_t0_	Number of opponents within the 1 m, 2 m, 5 m, and 10 m radius to pass the player at t0.
	Velocity PP_t0_	Instantaneous velocity of passing player at t0.
	Velocity nearest opp. PP_t0_	Instantaneous velocity of the nearest opponent to passing player at t0.
	Opponent angle	Angle (ɵ) between vectors $\vec{AB}$ and $\vec{AC}$ at t0. (cos ɵ = $\vec{AB}*\vec{AC}/|\vec{AB}|*|\vec{AC}|$).
	Foot /No foot	Indicates if the pass was performed with the foot or not (binary).
	One touch	Indicates if the pass was performed with the ball under the passer’s previous control or not (binary).
**Receiving player variables**	Nearest opp. RP_t0_	Nearest opponent to the receiving player at t0.
	Density RP_t0_	Number of opponents within the 1 m, 2 m, 5 m, and 10 m radius to pass the receiver player at t0.
	Velocity RP_t0_	Instantaneous velocity of the receiving player at t0.
	Nearest opp. RP_t1_	Nearest opponent to the receiving player at t1.
	Density RP_t1_	Number of opponents within the 1 m, 2 m, 5 m, and 10 m radius to the receiving player at t1.
	Velocity RP_t1_	Instantaneous velocity of passing receiver player at t1.
	Velocity nearest opp. RP_t1_	Instantaneous velocity of nearest opponent to the receiving player at t1.
	Displacement RP	Distance performed by the receiving player between t0 and t1.
**Ball trajectory variables**	Passing distance	Passing distance (vector modules $\vec{AB}$).
	Passing angle	Angle (ɵ) between vector $\vec{AB}$ and unit vector $\vec{v}$ oriented by the X axis of the pitch (ɵ = arctan).
	Ball velocity	Mean velocity estimated by the ratio of the passing distance to the time between t0 and t1.
	Ball progression	Variation of the ball’s position in relation to the X axis between t0 and t1.
	Outplayed opp.	Number of opponents between passing player at t0 and receiving player at t1 in relation X axis.
	Out ball angle	Angle (ɵ) between vectors $\vec{AB}$ and $\vec{DA}$. Calculation based on the angle between vectors (cos ɵ = $\vec{AB}*\vec{DA}/|\vec{AB}|*|\vec{DA}|$).
	Passing angle	Angle(ɵ) btw vectors ($\vec{AB}$) e unit vector oriented by the X axis of the pitch. (ɵ = arctan) (categorical).
	Passing accuracy	Indicates pass success and failure (binary).
**Pitch position variables**	Distance PP_t0_ to target	Distance btw passing player and target of opponent at t0.
	Distance RP_t0_ to target	Distance btw receiving player and target of opponent at t0.
	Distance RP_t1_ to target	Distance btw receiving player and target of opponent at t1.
	Opp. btw RP_t1_ and target	Number of opponents between target and receiving player in relation X axis at t1.

Abbreviations: opp = opponent; PP_t0_ = passing player at the time of the pass execution; RP_t0_ = receiving player at the time of the pass execution; PR_t1_ = receiving player at the time of the receipt of the pass; btw = between.

These variables were extracted from the 2D and scout matrix based mainly on two different moments, t_0_ and t_1_. t_0_ refers to the moment the *passing player* (PP) passes the ball, while t_1_ refers to the moment when the *receiving player (RP)* receives the ball. In the case of an unsuccessful pass, t_1_ refers to the moment when an opponent player intercepted the ball. In both moments, we recorded the 2D positional information (XY) of the passing player (PP_(t0)_) and the passing receiver player (PR_(t0)_ and RP_(t1)_), as well as all other players from both teams, team 1 (XY_1_, XY_2_,…, XY_14_) and team 2 (XY_15_, XY_16_,…, XY_29_). We consider the pass as a vector ($\vec{AB}$) originating from PP_(t0)_ (A) and ending in RP_(t1)_ (B), projected on the pitch). Another vector, $\vec{AC}$, was based on the PP_(t0)_ nearest opponent, i.e., with the origin in A and the extremity in the position nearest opponent (OP) to the passing player at t_0_ moment, OP_(t0)_ (C). The position variation of the PP also constituted an important vector, $\vec{DA}$, originating in PP_(t0-1)_ (D) and extremity in PP_(t0)_ (A).

**Fig 2:**
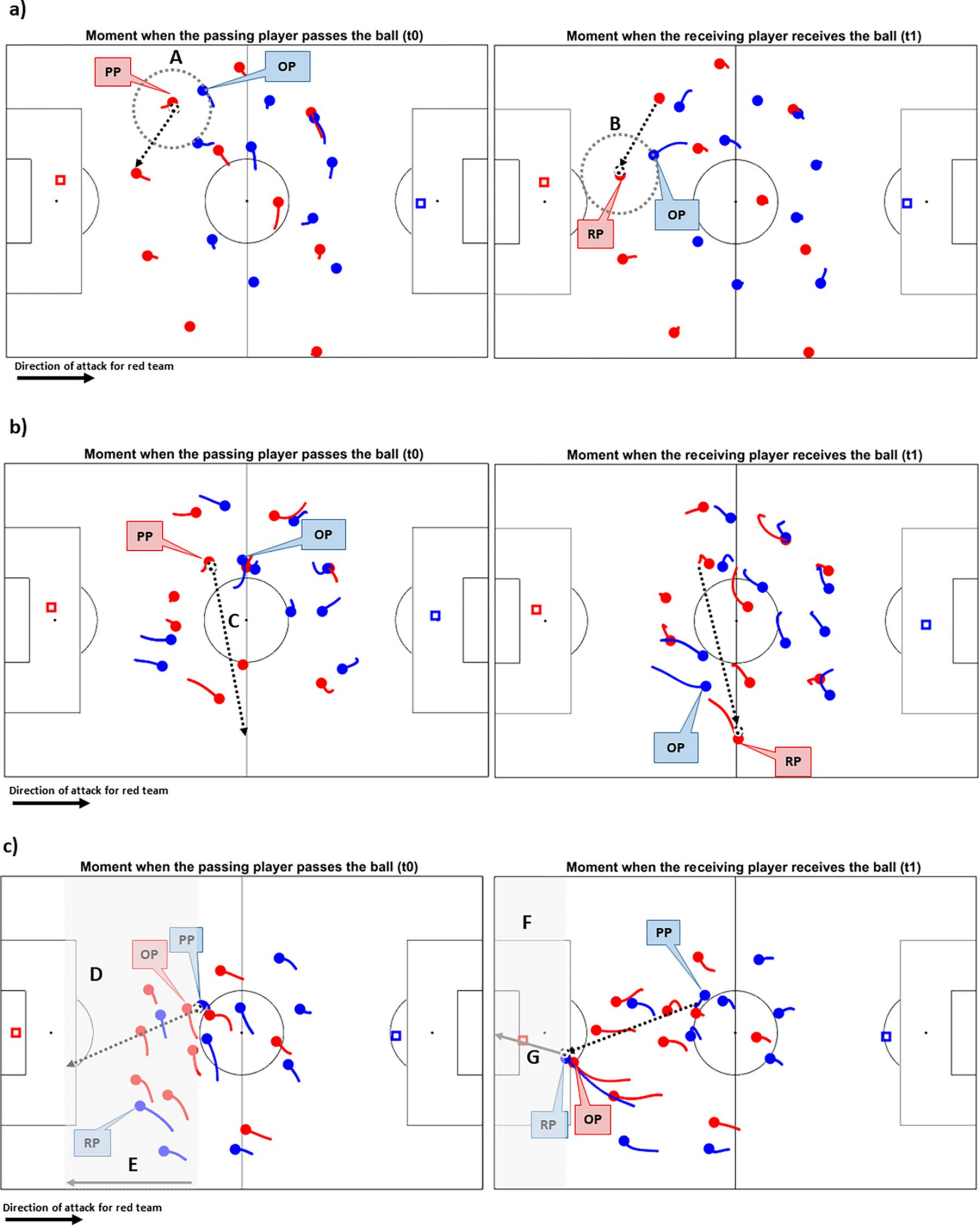
Ilustration of the variables in three different passes classified by the model. a) Example of low-DP. b) Example of medium-DP. c) Example of high-DP. Red team attacks to the right and blue team attacks to the left. Abbreviation: PP = passing player; RP = receiving player; OP = nearest opponent to the passing player and receiving player; A = 10 m radius to passing player at t_0_; B = 10 m radius to receiving player at t_1_; C = Passing distance; D = number of outplayed opponent (into light gray shaded area); E = Ball progress; F = opponent between PRt1 and target (into light gray shaded area); G = distance between receiving player and target of opponent at t_1_.

In cases where the player did not perform a pass successfully (for instance, this pass was intercepted by an opponent) the position of the possible receiver of the pass (expected receiver—ER) was estimated according to the equation $ER=\frac{distance}{shortest\;distance}.\frac{angle}{shortest\;angle}$, as proposed previously [19]. The ER position at the moment of the passing receipt, ER_(t1)_, was used as $\vec{AB}$ vector extremity when passes were considered as an unsuccessful action, and the calculation of other variables was based on the possible receiver position, both at t_0_ and at t_1_. This criterion was adopted considering that it is essential to observe characteristics of the PP intention to judge and determine its difficulty. Fig 2 illustrates the variables proposed in this study.

### Response variables (Labeling process)

Approximately 20% of the total samples (n = 465 passes) of the four matches combined were randomly separated for the passes labeling process. Two experts (researchers and coaches in soccer) performed, separately, the labeling process of passing through judgment. We presented to the experts only the concept of difficulty of the pass proposed in this study. Before the experts started judging the 465 passes, we presented 10 passes with different properties for them to adjust their own criteria when classifying the passes. For this study, passing difficulty was defined as the degree of technical and tactical demands that the passing player must complete the action successfully. Then, they watched videos of passes and assigned a classification for each event: class 1 (low difficulty), class 2 (medium difficulty), and class 3 (high difficulty). Experts could review the passes until they have a clear judgment. When they agreed about the classification of the passes, the judgments were validated. When there was disagreement, a third expert decided about the classification. Only the classification of the first two experts was considered for the agreement test.

The labels specified by the experts comprised the dependent variables of the model: *Y* = {*y*_1_,*y*_2_,…*y*_*n*_}.

### Dataset

The obtained predictor variables (X) and response variables (Y) were used to fit a supervised classification model. We modeled the problem considering the three classes. Thus, the dataset structure was composed of 465 events (passes), 35 predictor variables, and three response variables (three classes):

*X* = {*x*_1_,*x*_2_,…*x*_*n*_} where *x*_*i*_∈*R*^*m*^ and *m* = 35;

*Y* = {*y*_1_,*y*_2_,…*y*_*n*_} where *y*_*i*_ ∈ {low difficulty, medium difficulty, high difficulty}.

### Supervised learning algorithms

We compared eight supervised learning classifiers available in the scikit-learn v0.20.3 library [30], and traditionally used in classification problems: Random Forest (RF), Logistic Regression (LR), K-Nearest Neighbors (K-NN), Support Vector Machine (SVM), Linear Support Vector Machine (LSVM), Gaussian Naive Bayes (NB), Linear Discriminant Analysis (LDA), and MLP (Multilayer Perceptron). Data were processed in the *python 3*.*6* environment, following some steps until obtaining the classifiers performance indicators:

Pre-processing: Initially, we used approximately 20% of total passes of each game (total of the 4 games combined, which led to 465 passes) for the labeling process by experts. After this step, we used 465 labeled passes, predictor variables (X) and response variables (Y), in the training and testing process of the classifiers. We first separated the 465 passes into two subsets: training set (75%), and test set (25%). Then we split the training set into 5 folds. We used a stratified k-fold cross-validation to ensure the proportional distribution of samples into the three classes (low, medium, and high difficulty passes) within the training set and test set. We also scaled the predictor variables by applying the Z-score normalization.

Evaluation protocol: We used the grid search and k-fold cross-validation algorithms, with k = 5, to obtain the best parameters, using the training data only. More precisely, the protocol adopted in this study is composed of nested cross-validation. One cross-validation to divide the whole dataset into training and test, and another cross-validation, using only the training data, to find the best parameters for the classifiers. We first divided the training data into five-fold and then we perform a grid search over the folds to find the best model. Thus, we came up with one model for each fold of the outer cross-validation and we used the built model to predict data from testing for folds of that cross-validation.

Evaluation metrics: We adopted the use of balanced accuracy and f1-score to measure the performance of classifiers. The balanced accuracy in multiclass classification problems to deal with imbalanced datasets and is defined as the average of recall obtained on each class: ½ ((tp / (tp + fn) + (tn / (tn + fp)), where tp is the number of true positives and fp the number of false positives. The f1-score can be interpreted as a harmonic mean of the precision and recall, where an f1-score reaches its best value at 1 and worst score at 0: f1-score = 2 * (precision * recall) / (precision + recall). The precision is the ratio tp / (tp + fp) where tp is the number of true positives and fp the number of false positives. The recall is the ratio tp / (tp + fn) where tp is the number of true positives and fn the number of false negatives (30). We repeated the experiment ten times considering different seeds in order to measure aspects of generalization of the models and nested cross-validation for hyperparameter tuning. With this, we end up with 50 values of balanced accuracy and f1-score, i.e., ten values for each one of the five rounds of the cross-validation protocol.

The source code will be made and freely available for scientific purposes on GigHub repository (https://github.com/allansp84/pass-classification), upon acceptance of this article.

After identifying the classifier with the best performance among the eight models compared, the model chosen was used to predict the degree of difficulty of a set of unseen samples of passes (n = 2,057). The predicted sample plus the previously labeled sample comprised 2,522 passes. From this, it was possible to determine the success rate of the 2,522 passes separated by classes: successful rate in low difficulty passes, medium difficulty passes, and high difficulty passes. Then we used this information in the second part of this study, that is, to illustrate the use of the model with the best performance to distinguish players and positions.

### Application of the classification model with the best performance to distinguish players and positions when performing passes

This section refers to the second part of the study. We use principal component analysis (PCA) to identify the best passing player based on the percentage of successful passes in low, medium, and high classes. We used the PCA for three reasons: to explain the variability between the variable accuracy in low, medium, and high difficulty passes among players analyzed. To identify which of these three variables are most determinant to distinguish the best passing players and positions; and to rank the best passing players. For this purpose, players who did not perform at least 20 passes in total or at least five passes in each class were excluded. Therefore, we analyzed a total of 41 players categorized into five roles: external defenders (ED), central defenders (CD), defensive midfield (DM), offensive midfield (OM), and forwards (FW).

### Statistical analysis

Statistical analysis was divided into two parts according to the organization of this study. Firstly, we adopted the use of the weighted kappa method (*kw*) to measure the inter-rater agreement between the experts. We consider the following interpretation proposed by [31] to measure the strength of agreement based on Kappa values for categorical data: <0.00: Poor; 0.00–0.20: Slight; 0.21–0.40: Fair; 0.41–0.60: Moderate; 0.61–0.80: Substantial; 0.81–1.00: Almost Perfect.

In the first part of this study, to compare the classifiers performance, we used the Friedman test based on the average and standard deviation values of the balanced accuracy, k-fold (n = 5). The test was replicated ten times totaling 50 balanced accuracy values. When there was rejection of the null hypothesis, that is, equality between classifiers, a Nemenyi post hoc test was used to identify the differences. P value was used for comparison between all pairs. A p-value below 0.05 indicates that that comparison is statistically significant, that is, it is unlikely that two sets for error rates are samples from the same distribution. This step was performed in a *python 3*.*6* environment (library), and based on the proposal by [32] which suggests the use of non-parametric tests, especially those used in this study, for multiple comparison of machine learning data.

In the second part of this study, we use principal component analysis (PCA) to identify the best passing player based on the percentage of successful passes in low, medium, and high classes. The explained variance was based on the eigenvalues of each component. The correlation of the variables and each of the principal components was observed by the component matrix. The ranking of the best passing players was based on the scores of the first principal component (PC1). The PCA analysis was processed using IBM SPSS Statistics for Windows (Armonk, NY: IBM Corp).

## Results

The results obtained in the present study are presented according to the following sequence: characterization of the labeled sample (n = 465), comparison of the classifiers’ performance and application of the model with the best performance for match analysis.

The 465 passes were initially labeled into three classes by the experts. We observed an inter-rater agreement between the experts of 80.2%, which corresponds to 373 events out of the 465 passes. This result suggests a substantial agreement level (*kw* = 0.75) according to the interpretation [33]. The classification of the sample (465 passes) by the experts at the three classes was as follows: 56.6% for the low difficulty passes (class 1), 22.6% for the medium difficulty passes (class 2), and 20.9% for the high difficulty passes (class 3).

**Fig 3:**
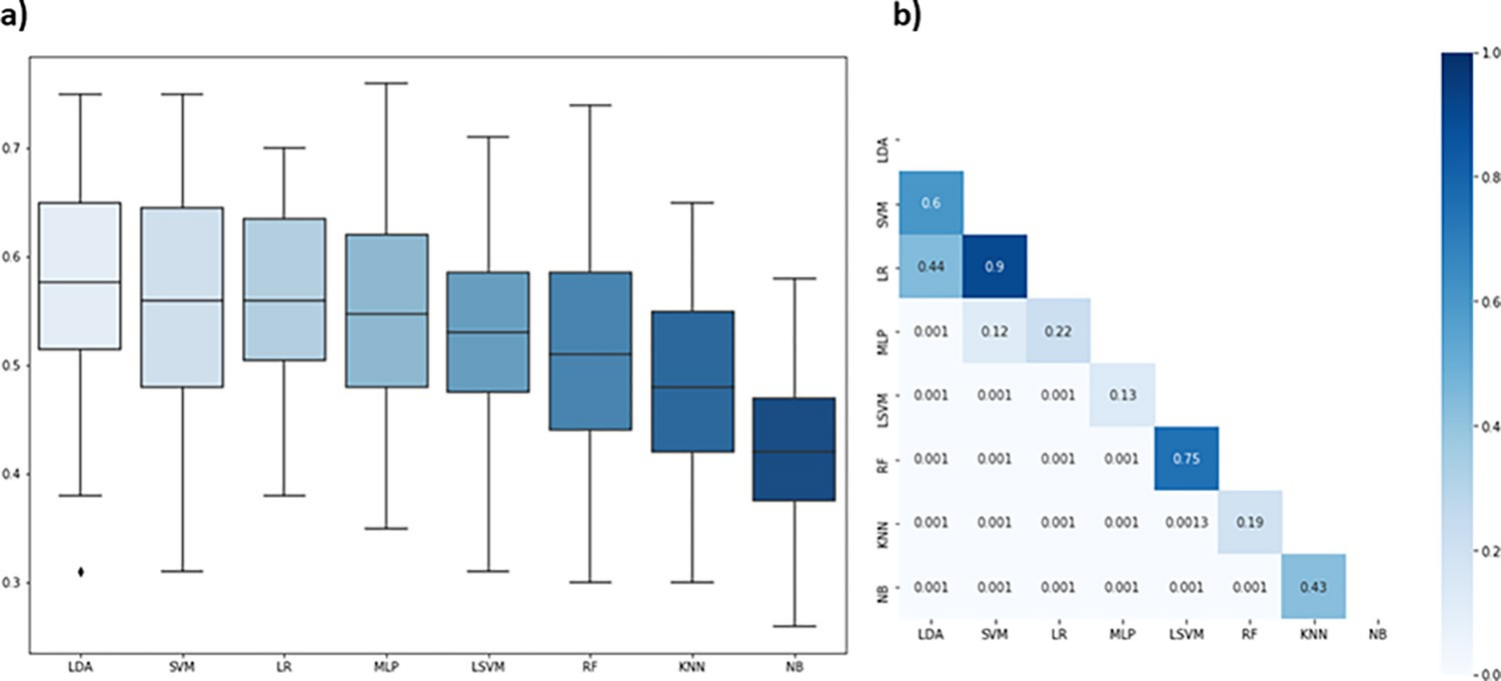
a) Comparison of performance based on balanced accuracy between machine learning classification in the condition 1 (three classes) using boxplot and Friedman statistical test. b) Pairwise comparison. The scale represents the p-value obtained through the Nemenyi post hoc test, also indicated into the squares (p-value below 0.05 indicates that that comparison is statistically significant). Abbreviation: SVM = Support Vector Machine, LR = Logistic Regression, LDA = Linear Discriminant Analysis, LSVM = Linear Support Vector Machine, MLP = Multilayer Perceptron, NB = Gaussian Naive Bayes, RF = Random Forest, K-NN = K-Neighbors Nearest.

Subsequently, the labeled dataset was used to train supervised learning classifiers. Table 2 summarizes the performance of the classifiers considering values of balanced accuracy and F1-score. The best performances observed were SVM (0.70 ± 0.04), LR (0.70 ± 0.05), and LDA (0.68 ± 0.05), which presented balanced accuracy values higher in relation to the other classifiers (Fig 3). In addition, SVM (0.71 ± 0.08) and LR (0.73 ± 0.07) presented higher F1-score values for the other classifiers, and there was no statistical difference between them. Among all the classifiers analyzed, we opted for SVM which, although there was no significant difference for the LR and LDA, was the one that reached the highest balanced accuracy value (Best accuracy = 0.88) in one of the rounds, that is, 88% correct when automatically classifying passes into three classes, low, medium, and high difficulty. We adopted the balanced accuracy values, that is, average percentage of correctness by classes based on k-fold cross validation. Our choice was based on the nature of the problem that focused on the model’s ability to correctly classify as many events as possible.

**Table 2:** Comparison of performance between machine learning classifiers.

Metrics	SVM	LR	LDA	L-SVM	MLP	NB	RF	K-NN
Bal. Acc.	0.70 ± 0.04	0.70 ± 0.04	0.68 ± 0.05	0.67 ± 0.04	0.64 ± 0.03	0.62 ± 0.03	0.62 ± 0.03	0.58 ± 0.05
Best Acc.	0.88	0.87	0.80	0.78	0.85	0.75	0.74	0.82
F1-score	0.71 ± 0.08	0.73 ± 0.07	0.75 ± 0.07	0.73 ± 0.07	0.72 ± 0.06	0.71 ± 0.07	0.72 ± 0.05	0.70 ± 0.07

Abbreviations: RF = Random Forest, LR = Logistic Regression, K-NN = K-Nearest Neighbors, SVM = Support Vector Machine, LSVM = Linear Support Vector Machine, NB = Gaussian Naive Bayes, LDA = Linear Discriminant Analysis and MLP = Multilayer Perceptron, Bal. Acc. = Balanced Accuracy, Best Acc. = Best Accuracy.

**Fig 4:**
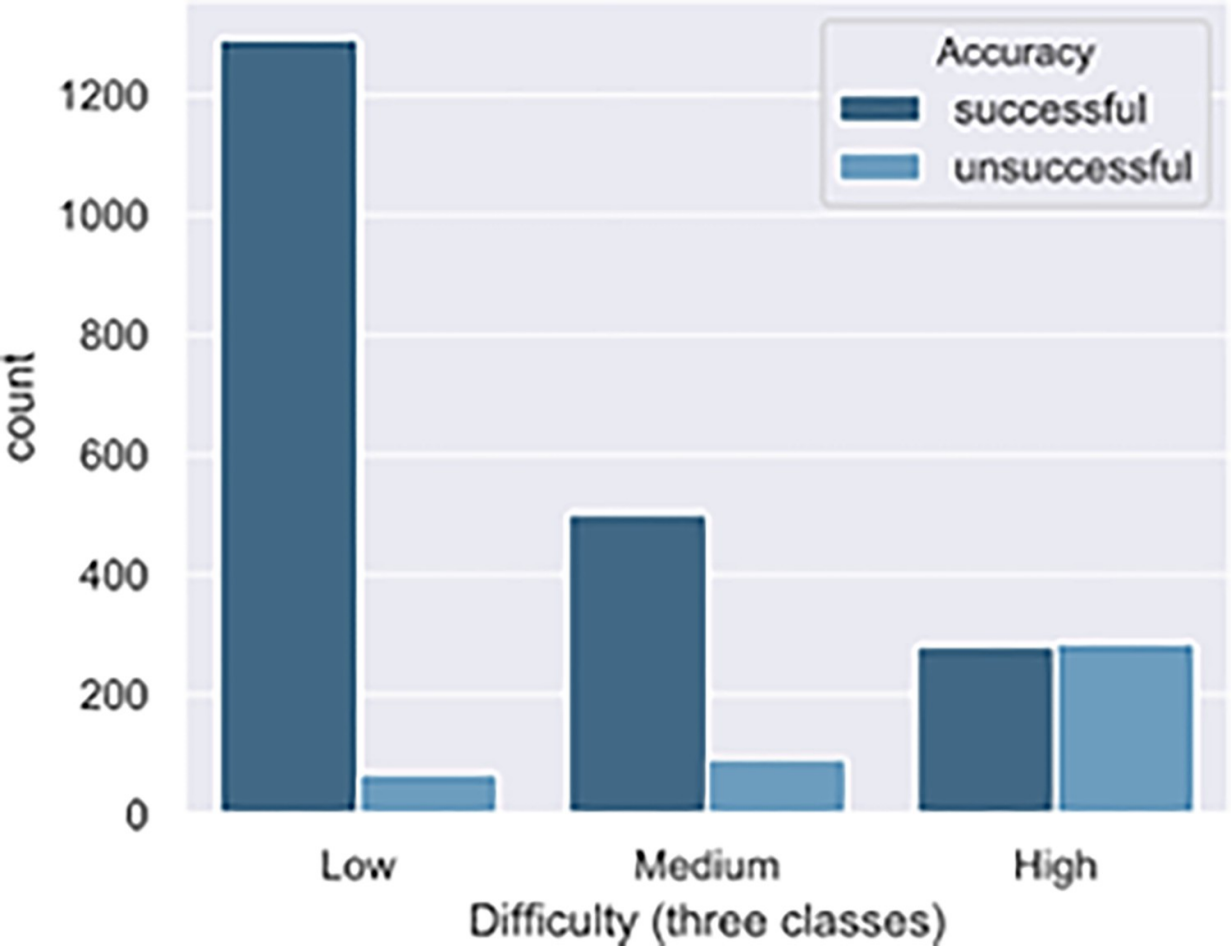
Total sample (n = 2,537) classified into three classes according to passing difficulty and accuracy.

After determining the classification model, we predicted all unlabeled passes (2,057), totaling 2,522 passes. The total sample was classified as 1,360 low difficulty passes (53.9%), 594 medium difficulty passes (23.6%) and 568 high difficulty passes (22.5%). We identified that the percentage of successful passes were 94.9, 84.0, and 49.3 for low difficulty passes, medium difficulty passes, and high difficulty passes respectively, Fig 4.

In the second step of this study, we illustrate the use of the best-performing classifiers in the analysis of players according to their passing performance. In this study, we analyzed the percentage of successful passes for each player for the different pass categories, which will be referred to as low difficulty pass accuracy, medium difficulty pass accuracy, and high difficulty pass accuracy. In an exploratory analysis, we have observed that players achieve different success rates in low, medium, and high difficulty passes. For example, player 16 achieved 77.8%, 84.6%, and 100.0% accuracy in low, medium, and high difficulty passes, respectively, while player 68 achieved 95.5%, 0.0%, and 20.0% (Table 3).

**Table 3:** Ranking of the best passing players ordered from the principal component 1.

Ranking	Player	Position	Accuracy (%)			Scores		
			Low	Medium	High	PC1	PC2	PC3
1°	16	OM	77.8	84.6	100.0	2.02	-2.32	0.99
2°	27	DM	97.8	94.7	100.0	1.82	0.55	1.62
3°	59	FW	81.8	91.7	83.3	1.63	-1.56	0.23
4°	24	ED	100.0	85.7	85.7	0.98	0.69	1.58
5°	13	OM	87.5	100.0	55.6	0.88	-0.52	-0.93
6°	18	FW	94.4	100.0	60.0	0.83	0.38	-0.38
7°	55	DM	96.4	94.4	66.7	0.79	0.49	0.24
8°	72	DM	93.8	100.0	57.1	0.76	0.30	-0.53
9°	58	OM	100.0	88.9	75.0	0.76	0.81	1.02
10°	74	OM	100.0	84.6	77.8	0.69	0.70	1.32
:								
37°	17	DM	96.2	91.7	16.7	-0.89	0.58	-1.63
38°	43	FW	100.0	75.0	37.5	-0.93	0.63	0.16
39°	20	FW	100.0	57.1	50.0	-1.17	0.18	1.46
40°	42	ED	88.9	66.7	28.6	-1.21	-0.99	-0.42
41°	68	CD	95.5	0.0	20.0	-4.05	-1.60	2.60

Abbreviations: PC1 = first principal component, PC2 = second principal component, PC3 = third principal component. ED = external defenders, CD = central defenders, DF = defensive midfield, OM = offensive midfield, FW = forwards. Low = low difficulty passing, Medium = medium difficulty passing, High = high difficulty passing.

**Fig 5:**
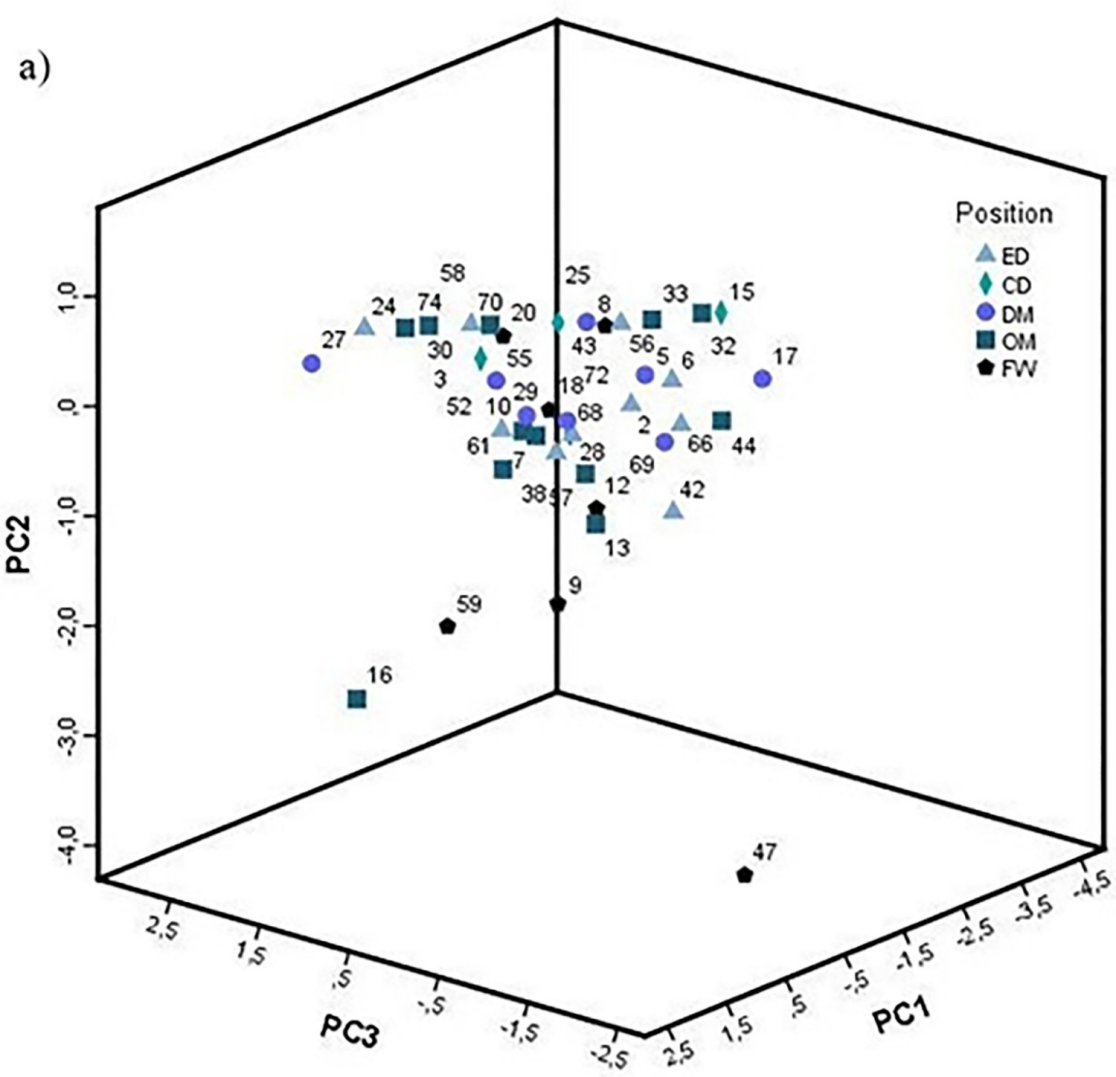
Three-dimension plot of the principal component analysis (PCA) of 41 players based on accuracy in low, medium and high difficulty passes, categorized by their specific positions. Abbreviations: PC1 = principal component, PC2 = principal component, PC3 = principal component. ED = external defenders, CD = central defenders, DF = defensive midfield, OM = offensive midfield, FW = forwards.

The challenge, therefore, was to explain the variance of these three variables (low, medium, and high difficulty passes accuracy) and determine who are the best passing players. For this purpose, these three variables were used as an input for principal component analysis (PCA). The PCA revealed three main components (PC1, PC2, and PC3) that together explain 100% of the total sample variance. PC1, which explains 41.3% of the variance, showed a higher correlation with high difficulty passes accuracy (0.80), followed by medium difficulty passes accuracy (0.73), and low difficulty passes accuracy (0.24). PC2, which explains 33.9% of the variance, showed a greater correlation with low difficulty passes accuracy (0.93), followed by medium (0.38), and high difficulty (0.07). And PC3 explains another 24.7% of the variance showed a higher correlation with the accuracy in high difficulty passes (0.59), followed by the accuracy in medium (-0.55), and low difficulty passes (0.27). Fig 5 show the position of each player categorized by specific positions in relation to PC1, PC2, and PC3 based on their scores. When we ordered the players from the PC1 scores, we obtained the ranking of the best passing players (Table 3).

## Discussion

The aim of this study was twofold. The first one was to classify automatically the degree of passing difficulty in soccer matches using machine learning classifiers. The support vector machine (SVM), a non-linear model, proved to be the best prediction model based on machine learning techniques capable of classifying passes with different degrees of difficulty. The SVM presented a mean of balanced accuracy of 0.70, that is, it suggests that the model has a 70% chance to correctly classify a pass in professional soccer matches in low, medium and high difficulty, based on the predictor variables proposed in that study. The SVM reached a balanced accuracy of 88% in their best performance. Some considerations must be made based on the results found.

It was clear that this is an unbalanced classification problem. The sample labelled by experts was distributed with 56.6% for the low difficulty passes (class 1), 22.6% for the medium difficulty passes (class 2), and 20.9% for the high difficulty passes (class 3). For this we chose balanced accuracy as the ideal metric to compare classifiers. The balanced accuracy considers the average of the ratio between true positives plus true negatives for the total sample in each class, that is, the maximum capacity of the model to classify correctly, considering each class, thus avoiding overestimating the classifier’s performance. The experts agreed in 80.2% of the cases when labeling the 465 events in three classes. This percentage suggests the subjective nature of the problem, which makes it difficult to achieve very high accuracy values.

Similar research has also been using passing prediction models in professional soccer matches. The passing ability model is based on the probability that each pass is successful, given information on the environment in which the pass was made and the identity of the player making the pass [34]. Mchale & Relton [28] aimed to identify key players using network analysis and difficulty passes, but defined difficulty as a synonym for importance and also assumed as a criterion the probability to complete the pass. Power et al. [19] proposed a logistic regression model to assess the risk and advantage of the pass. The risk is conditioned by the likelihood that the player will successfully make the pass given a player has possession of the ball and the advantage the likelihood that the pass made will result in a shot within the next 10 seconds. They assign higher values for passes less likely to be completed. The present study proposed a set of variables different from the others, aiming to contemplate technical and tactical attributes of the match, i.e., observing and judging the degree of difficulty of each event based on the proposed concept.

Other recent studies have also had the challenge of improving information about the passes in soccer matches, through metrics to measure effectiveness [22,24,29], probabilistic models [20], indexes [9], among others. In the study that most resembles ours, Chawla et al. [21] obtained 90.0% accuracy to classify quality of the pass in good, ok, and bad, using a logistic regression model. In their work, unsuccessful passes were excluded. These results are higher than the present study, but it is not clear if the reported accuracies refer to a balanced accuracy score. Recall that ours refer to an unbalanced scenario analysis. As a general idea, the studies start from the same principle, that is, to assign greater weight in the effectiveness in performing more difficult passes, either through regression where the outputs are continuous values, or classification where the outputs are categorical. The present research brings some fundamental differences. The concept of passing difficulty was originally proposed and is essential to our problem. The focus of the experts when labeling passes was centered in the degree of technical and tactical demands that the passing player must complete the action successfully. Furthermore, in our conception, there is a difference between difficulty and quality or advantage of the passes. We focused on the difficulty because we wanted to analyze the player’s ability to perform passes relativizing by the degree of difficulty.

Normally, in professional first division matches, players presented an average success rate of 84.3%, as an English Premier League games [19]. In our sample, the first division of Brazilian soccer, we observed a success rate of 82.3%. When we observed the percentage of successful passes in each class, we found that high difficulty passes had a success rate of 49.3% only, followed by 84.0% for medium difficulty passes, and 94.9% for low difficulty passes (Fig 4). These data demonstrate the importance of analyzing the success rate in passes with different levels of difficulty.

In the first application from the complete sample, 2,522 passes were classified within the three classes and categorized into successful and unsuccessful passes. We sought to characterize each player and position, and to discriminate the best passing players. The exploratory analysis from frequency of occurrence showed that the proportion of low, medium, and high difficulty passes is different between positions. A higher proportion of high difficulty passes for forwards (FW) and offensive midfielder (OM) in relation to the other positions. However, it was necessary to analyze the performance of each player and position for each class. Therefore, these three variables, accuracy in low, medium, and high difficulty passes, were used as inputs for principal component analysis (PCA). The PCA revealed three principal components that together explained 100% of the variance contained in the three predictor variables. PC1, which explains most of the variance, showed a higher correlation with the variable accuracy in high and medium difficulty passes. This finding suggests that it is more important to consider the player’s ability to complete high and medium difficulty passes than low difficulty passes. When we observed the players’ ranking from the PC1 score, the best ranked players, players 16 and 27, showed 100.0% efficiency in high difficulty passes. On the other hand, the last ranked player, 68, although he showed 95.5% efficiency in low difficulty passes, showed only 20.0% efficiency in high difficulty passes.

The present study aimed to improve the level of information on the most frequent and determinant technical-tactical action in soccer matches. Classifying the pass in different degrees of difficulty proved to be important when analyzing the efficiency relativized by the complexity of the performed action. The player ability in performing more difficult tasks, in this case the pass, was determinant when discriminating players and positions, and it can also contribute to discriminate teams, analyze offensive sequences, among other applications.

## Conclusion

In summary, the SVM classifier showed better performance among the other classifiers when classifying passes in low, medium and high difficulty based on the predictor variables. We applied the classification model, SVM, to predict a new sample of passes. Then, we identified through the principal components analysis that the efficiency in performing high and medium difficulty passes is more determinant to distinguish players and positions, that is, the best passing players were those who had the highest percentage of successful passes in high and medium difficulty, respectively. The proposed model improved the level of information about passing actions, which is the most frequent and determinant for performance in soccer matches. In addition, the model can be applied in other analyzes, such as offensive sequences analysis. We believe that by classifying passes by their difficulty, we could compare successful and unsuccessful offensive sequences (which end in shots), identifying the passes characteristics in both cases. That is, what passing characteristics are necessary for a successful attack. Furthermore, the model would allow for distinguishing the best passing players, and not players with a high rate of successful passes, as traditionally used.

The present study analyzed a sample of passing events obtained from matches in the Brazilian men’s football championship. Future studies could expand the number of matches, passing events, testing other leagues and even women’s football leagues. This could contribute to further improving the performance of the passing difficulty classification model in football matches.
